# Production and Utilization of Fermented Rice Bran as Animal Feed

**DOI:** 10.1111/asj.70037

**Published:** 2025-02-13

**Authors:** Jamal James D. Manlapig, Hiroki Matsui

**Affiliations:** ^1^ Department of Animal Science, College of Agriculture Central Luzon State University Science City of Muñoz Nueva Ecija Philippines; ^2^ Graduate School of Bioresources Mie University Tsu Mie Japan

**Keywords:** fermentation, fermented rice bran, lipase activity, microorganisms

## Abstract

Rice bran, a byproduct of milling rice ( *Oryza sativa* ), is widely used in Asian preserved foods and the feed industry. Rich in carbohydrates, protein, fat, and various bioactive compounds, it offers significant growth and health benefits. It is commonly used as a replacement for cereals and grains as an energy source in the diet. However, its potential as animal feed is constrained by antinutritional factors and susceptibility to rancidity due to lipase activity. Recently, there has been a growing interest in fermentation to enhance rice bran's nutritional profile. It has been confirmed that fermenting rice bran could increase the proportion of crude protein and fat. Moreover, feeding fermented rice bran (FRB) improves the growth performance of food‐producing animals by increasing efficiency. These results, along with their beneficial effect on gut microbiota, may improve the overall health of food animals. In this review, we aimed to summarize the latest research on the fermentation of rice bran, focusing on the role of microorganisms and the factors influencing the quality and nutritional value of FRB. Additionally, we will explore the impact of FRB on nutrient composition and animal performance, highlighting its potential as a valuable feed ingredient.

## 1 Introduction

The quality and availability of feed resources greatly affect the cost associated with feeding. Feed cost amounts to 70% of the total production cost of livestock operation (Makkar 2018) due to the high amount of cereals and grains in the diet. Thus, in recent years, the use of non‐conventional feed resources and industrial and agricultural by‐products such as brans, pulps, brewers, spent grains, and acacia pods, among others, as alternative protein and energy sources in the diet have been extensively reviewed (Crisostomo et al. 2022; Sugiharto 2022).

Rice bran is a by‐product of rice milling, comprising about 8%–9% of rough rice's total weight. In 2019, global rice bran production was estimated to exceed 60 million metric tons. Approximately 90% is utilized as feed for livestock and poultry, while the remainder is processed for rice bran oil extraction (Schramm et al. 2007).

Rich in protein, fatty acids, dietary fiber, and minerals (McCaskill and Zhang 1999), rice bran has significant potential as a feed ingredient. It also contains substantial amounts of vitamins, amino acids (Younas et al. 2011), bioactive compounds (Ardiansyah 2021), and natural antioxidants (Godber and Wells 1994), which can enhance dry matter intake, promote body weight gain (Criscioni and Fernández 2016), and potentially reduce methane emissions in vitro (Manlapig et al. 2024; Cao, Horiguchi, and Takahashi 2009). Despite these advantages, the inclusion of rice bran in animal feed remains low due to the presence of antinutritional factors such as phytic acid, trypsin inhibitors, oxalates, saponins, and tannins (Irakli, Lazaridou, and Biliaderis 2020), which can negatively impact digestibility and nutrient bioavailability. Additionally, the high lipid content makes rice bran susceptible to hydrolytic rancidity when exposed to active enzymes, further complicating its storage and use (Liu et al. 2023). Addressing these challenges is essential to unlock the full potential of rice bran in animal nutrition.

Several strategies can be employed to address the challenges associated with rice bran, including cooking, sun‐drying, and fermenting. Cooking and sun‐drying can help reduce moisture content and deactivate certain antinutritional factors, such as phytic acid and trypsin inhibitors (Samtiya, Aluko, and Dhewa 2020; Sharma, Chauhan, and Agrawal 2004). However, these methods may not fully enhance the bioavailability of nutrients. Fermentation, on the other hand, offers a unique approach that not only reduces antinutritional compounds but also enriches rice bran with beneficial microorganisms, enzymes, and metabolites that improve digestibility. This process can enhance the availability of essential amino acids and vitamins, making fermented rice bran (FRB) a more nutritious feed option for livestock. This review will delve into the potential of fermentation as a viable solution to the limitations of rice bran, examining its impact on nutrient composition, palatability, and overall animal performance. By optimizing fermentation conditions and selecting appropriate microbial strains, we can unlock the full potential of rice bran as a high‐quality feed ingredient.

## 2 Production of Fermented Rice Bran

Fermentation is a chemical process in which carbohydrates are utilized and broken down by microorganisms anaerobically. Traditionally, this technique has been used for food preservation. In the agriculture industry, there are two major methods widely used: submerged fermentation (SmF) and solid‐state fermentation (SSF). SmF is a technique used to grow microorganisms in a liquid medium to produce a variety of biochemical products (mostly enzymes) (Trivedi, Bhonsle, and Atray 2020), while SSF is the process of fermentation with almost no free water and the process that best mimics the natural habitat of most filamentous fungi (Hansen et al. 2015), suitable for the production of food, feeds, and fuels (Navarro‐Pineda et al. 2016).

FRB is produced by fermenting raw or defatted rice bran with or without the use of microbial inoculants. SSF is the most often used method employed in fermenting rice bran. It offers numerous advantages over other types of fermentation, such as low‐cost media/substrate, simple technology, and higher productivity, and it resembles the natural habitat of several microorganisms (Couto and Sanromán 2006). Microorganisms such as bacteria and fungi are commonly used as inoculum in this technique that produces a variety of enzymes (cellulase, pectinases, xylanase, chitinase, lipase, and β‐galactosidase) able to break complex carbohydrates, thus increasing the bioactive compounds (Ardiansyah 2021), nutritive value (Manlapig, Ban‐Tokuda, and Matsui 2023; Omarini et al. 2019) and improve sensory profile (Schmidt et al. 2014) of rice bran.

## 3 Factors Affecting the Quality of Fermented Rice Bran

In determining the quality of the end product, it is important to consider the following: source of the substrate; the species and abundance of microorganisms present in the raw material and inoculant used; and parameters during fermentation, particularly the length of incubation and temperature (Missotten et al. 2015).

### 3.1 Substrate

There are more than 127,000 cultivars of rice in the world, and 40,000 of these are grown commercially, mostly in Asia (Awan et al. 2017). They can be classified into aromatic, non‐aromatic, non‐glutinous rice, and glutinous or sticky rice. Among these, it can be further classified into pigmentation (color) and grain size and length (long, medium, and short). Pigmented varieties of rice attribute their coloration to the high amount of anthocyanin compounds present in its pericarp and aleuronic layers (Shao et al. 2018; Huang and Lai 2016). Accordingly, the nutritional composition of rice grain and bran differs from one variety to another.

The proximate composition of nutrients in white rice and colored rice bran is shown in Table 1. Rice bran, regardless of variety, is considered an energy source containing a high amount of carbohydrates and fat. Total carbohydrates in rice bran account for more than 60% on a dry matter (DM) basis (Kalschne et al. 2020; Huang and Lai 2016). The lipid content of pigmented rice varieties was higher than that of the white rice varieties. Consequently, the protein content in white varieties was higher than in pigmented varieties. Lipid and protein content in a feed ingredient is directly associated with its quality, especially in agro‐industrial byproducts such as rice bran used in non‐ruminant feeding.

**TABLE 1:** Proximate composition of nutrients (%) white and colored rice bran.

Variety	Lipid	Protein	Ash	Total carbohydrate
White				
BRS AG	10.40 ± 0.98	14.14 ± 0.10	5.40 ± 0.55	69.72 ± 0.77
BRS Pampa	10.87 ± 0.81	13.26 ± 0.97	7.47 ± 0.78	68.30 ± 1.14
BRS 358	14.99 ± 0.52	17.50 ± 0.16	7.37 ± 0.41	60.14 ± 1.01
Pigmented				
HB	13.54 ± 0.17	11.28 ± 0.83	6.13 ± 0.04	69.12 ± 0.95
WB	13.03 ± 0.22	12.49 ± 0.04	5.99 ± 0.07	68.59 ± 0.15
TB	12.47 ± 0.66	11.77 ± 0.00	5.07 ± 0.08	71.04 ± 0.15
HR	14.07 ± 0.42	10.64 ± 0.13	8.12 ± 0.08	67.43 ± 0.02
GR	16.34 ± 0.37	10.71 ± 0.72	8.58 ± 0.04	64.37 ± 1.03
TR	17.34 ± 0.53	9.64 ± 0.31	7.13 ± 0.03	66.18 ± 0.36

*Note:* Adapted from Huang and Lai (2016) and Kalschne et al. (2020).

Abbreviations: BRS, rice cultivars developed by Brazilian Agricultural Research Corporation (Embrapa); GR, Guangfu red rice; HB, Taibalang black waxy rice; HR, Taibalang red waxy rice; TB, black rice Thailand; TR, red rice Thailand; WB, black rice western Taiwan.

### 3.2 Microorganisms

The fermenting microorganisms, along with the composition of substrates, are the major factors that influence the quality of fermented products (Sharma et al. 2020). During fermentation, microorganisms transform raw materials by altering the proximate composition of nutrients. Moreover, several studies show that microorganisms could increase the bioavailability of nutrients by reducing the amount of anti‐nutritional factors (Shuvo et al. 2022; dos Santos Oliveira et al. 2011) and increasing the total phenolic and antioxidant activity (Ardiansyah 2021; Alauddin et al. 2016; Abd Razak et al. 2015) of rice bran after fermentation, thereby improving its overall feed quality.

The amount of naturally occurring microorganisms and the starter culture added to the rice bran determine the rate and extent of fermentation. Inoculation of rice bran with starter culture has been a common practice to speed up the process and increase the production of beneficial products. The type and concentration of inoculant directly affect the amount of lactic acid produced during fermentation. The immediate increase in the concentration of lactic acid during the fermentation process leads to a rapid decrease in pH. This rapid drop in pH can inhibit the growth of unwanted microorganisms such as *Salmonella* spp. and *Escherichia coli* (Missotten et al. 2010).

Lactic acid bacteria (LAB) and fungi are the most used inoculants in the production of FRB for their ability to produce enzymes that breakdowns complex compounds, increase nutrient availability, increase total phenolic activity, increase antioxidant activity, and reduce antinutritional factors (Table 2).

**TABLE 2:** Effect of fermentation and inoculant on the nutritional composition of rice bran.

Inoculant (microbes)	Fermentation (h)	Results	Reference
Bacteria			
*Bacillus amyloliquefaciens*	72	Increase crude protein and gross energy content	Supriyati et al. 2015
		Decrease crude fiber content	
LAB (*Lactobacilus bulgaricus, Streptococus thermophilus* and *Lactobacilus acidophilus*)	0, 5, 10, and 15 weeks	Decrease phytic acid and crude fiber	Sulendre and Pamulu 2021
*Lactiplantibacillus plantarum* EM	48	Strong antimicrobial activity	Moon and Chang 2021
		Decrease phytate level	
*Limosilactobacillus equigenerosi*, *Ligilactobacillus equi*, and mixture	96, 168, and 240	Increase crude protein, ash, lipid, fiber, and gross energy content	Manlapig, Ban‐Tokuda, and Matsui 2023
		Decrease nitrogen‐free extracts	
Fungi			
*Aspergillus flavus* PHY168	48, 72, and 96	Increase crude protein, ether extract, ash, and phosphorus content	Ahmad et al. 2019
		Decrease DM and fiber	
*Aspergillus niger*	0, 24, 48, and 72	Increase DM and diluted protein	Hardini 2010
		Decrease organic matter	
*Monascus pilosus* KCCM60084	240	Increase total flavonoid	Cheng et al. 2016
*Pleurotus sapidus*	96, 144, and 240	Increase crude protein, ash, and total carbohydrates	Omarini et al. 2019
		Decrease lipid content	
*Rhizopus oligosporus*	72	Increase in total phenolic content and antioxidant activity	David et al. 2019
*Rhizopus oligosporus, Rhizopus oryzae* and mixture	48, 72, and 96	Increase in total phenolic content and antioxidant activity	Ardiansyah 2021
*Rhizopus oligosporus* (strain F0020), *Monascus purpureus* (strain F0061) and mixture	288	Increase in total phenolic content and antioxidant activity	Abd Razak et al. 2015
*Rhizopus oryzae* CCT 7560	24	Increase in ash content, dietary fiber, and protein content	Kupski et al. 2012
		Decrease in lipid content	
*Rhizopus oryzae*	96	Increase ash content, dietary fiber, protein, and amino acid	Oliveira et al. 2010
		Decrease in water content, lipid, and phytic acid	
*Rhizopus oryzae* CCT 7560	96	Increase in phospholipid and unsaturated fatty acid	dos Santos Oliveira et al. 2011
		Decrease total lipid and saturated fat	
*Rhizopus oryzae*	120	Increase in total phenolic content	Oliveira et al. 2012
*Rhizopus oryzae* CCT 7560	96	Increase in dietary fiber, ash content, protein, lipid, and phenolic content	Ribeiro et al. 2017
*Saccharomyces boulardi*	24	Increase in bioactive compounds and decrease in lymphocyte B cell	Ryan 2011
*Saccharomyces cerevisiae*	24	Decrease fiber and phytate‐P level	Azrinnahar et al. 2021
Mix cultures			
*Aspergillus kawachii +* LAB ( *Lactobacillus brevis* , *Lactobacillus rhamnosus* , *Enterococcus faecium* )	44 + 24	Increase in dietary fiber, lipid content, and total phenolic content	Alauddin et al. 2016
Rumen fluid	12	Decrease crude fiber, neutral detergent and acid detergent fiber, cellulose, and hemicellulose	Debi, Wichert, and Liesegang 2022
Rumen fluid	48	Increase crude protein	Shuvo et al. 2022
		Decrease fiber and phytate‐P content	

### 3.3 Fermentation Parameters

Other than substrate and microorganisms, the quality of FRB is also affected by temperature and incubation length (hours). The effect of fermentation temperature (25°C–35°C), yeast concentration (1%–5%), and fermentation time (10–24 h) on the protein yield of FRB was investigated by Chinma, Ilowefah, and Muhammad (2014). They reported that optimum conditions for yeast pretreatment of rice bran for protein extraction were achieved at 30°C for 17 h using a 3% yeast concentration to obtain a protein yield of 23.37%. In addition, incubation temperatures above 30°C resulted in the reduction of the protein concentrate extracted. This result was associated with *Saccharomyces cerevisiae's* optimal fermentation temperature, which was found to be between 25°C and 28°C (Redón et al. 2011; Attfield 1997)**.**

Hardini (2010) studied the effect of incubation length on the nutritive value of FRB mixed with *Aspergillus niger*. The results show that DM content, diluted protein, and N retention of FRB were increased in response to the increasing length of incubation. Moreover, Omarini et al. (2019) also reported that the protein content of rice bran fermented with *Pleurotus sapidus* significantly increased after 10 days of fermentation compared to 4 and 6 days at 12.8 ± 0.6, 10.8 ± 1.3, and 9.7 ± 0.8, respectively. Similar results were reported with *Limosilactobacillus equigenerosi* and *Ligilactobacillus equi* FRB (Manlapig, Ban‐Tokuda, and Matsui 2023).

## 4 Effect of Fermentation on Rice Bran Composition

The composition of FRB results from the combination of the chemical composition of the substrate, the interaction of the starter culture used with the substrate, and the secondary substances produced during the fermentation process.

Several studies consider SSF as a means of improving the nutrient value of rice bran as feed, and results show a significant increase in the protein content of FRB (Table 2). Ribeiro et al. (2017), Kupski et al. (2012), and Oliveira et al. (2010) reported that the use of *Rhizopus oryzae* CCT 7560 in the production of yielded a significant increase in the protein content by 141% (96 h), 49% (120 h), and 40% (120 h), respectively, after fermentation. Moreover, the digestible amino acid (AA) also increased by 27.6% in the digestibility of produced proteins (Oliveira et al. 2010). Similar findings were reported in rice bran fermented with *Aspergillus flavus* (Ahmad et al. 2019), *A. niger* (Hardini 2010), *Bacillus amyloliquefaciens* (Supriyati et al. 2015), and rumen fluid (Shuvo et al. 2022). The increase in protein was due to the secretions of microbial proteins like enzymes and other nitrogenous components. Some species of fungi have been reported to increase the protein contents of agro‐industrial raw materials after fermentation (Ravindra 2000). Moreover, microorganisms have been used extensively in the production of protein from agro‐industrial waste, mainly for animal feed (Kupski et al. 2012). They utilize carbohydrates as a source of energy and bio‐converted them into microbial proteins through intermediary metabolisms (Rajesh and Raj 2010).

There are mixed results regarding the effect of fermentation on the fibrous component of rice bran. Alauddin et al. (2016) prepared FRB by dual fermentation using fungi (*Aspergillus kawachii*) for 44 h and then LAB for 24 h (Table 2). They reported a significant increase in the dietary fiber of fermented than unfermented at 4.4% and 22%, respectively. Similar results were observed with rice bran fermented with *R. oryzae* CCT 7560 (Ribeiro et al. 2017; Kupski et al. 2012; Oliveira et al. 2010). They reported that FRB's crude fiber (CF) content continuously increases, and the highest concentration was recorded after 96 h of fermentation. This fiber increase comes from fungal polysaccharides produced during the fermentation process (Oliveira et al. 2010), which are commonly composed of cellulose and chitin (Griffin 1994). Conversely, Ahmad et al. (2019) and Azrinnahar et al. (2021) reported a decrease in CF content after fermentation with *A. flavus* and *S. cerevisiae* . This agrees with the results of Shuvo et al. (2022) and Debi, Wichert, and Liesegang (2022), where FRB's fiber component decreases after incubation with rumen fluid. The CF reduction was associated with fibrolytic enzymes in *A. flavus* , *S. cerevisiae* , and microorganisms in the rumen fluid that degrades complex carbohydrates into simple sugars.

Similar findings have been reported regarding the lipid content in FRB. Specifically, an increase in lipid content was observed in rice bran fermented with *A. flavus* (Ahmad et al. 2019) and through dual fermentation with *A kawachii* and LAB (Alauddin et al. 2016). Also, lipid content was increased in rice bran fermented with *Limosilactobacillus equigenerosi, Ligilactobacillus equi*, and their combination (1:1) (Manlapig, Ban‐Tokuda, and Matsui 2023). These results highlight the potential of fermentation techniques to enhance the lipid profile of rice bran, which can contribute to its nutritional value as an animal feed ingredient. Conversely, reduction was also reported with rice bran fermented with *R. oryzae* CCT 7560 (Ribeiro et al. 2017; Kupski et al. 2012; Oliveira et al. 2010). The lipid reduction was the result of fungal use in the production of their cell membrane composed of phospholipids, which was commonly observed in this species (Oliveira et al. 2010).

Ash content was significantly increased by the addition of fungi during fermentation, and a higher concentration was recorded at 72–96 h of incubation (Ahmad et al. 2019; Ribeiro et al. 2017; Kupski et al. 2012; Oliveira et al. 2010), which was attributed to the inherent ash content of fungi. In addition, the phytate contents were also reduced in FRB than in unfermented rice bran (Shuvo et al. 2022; Azrinnahar et al. 2021; Moon and Chang 2021; Oliveira et al. 2010). These results were probably due to the high phytase activity of *A. flavus* and *R. oryzae* CCT 7560 (Ardiansyah 2021; Oliveira et al. 2010). Phytase is an enzyme that accelerates the removal of phosphate from either phytic acid or phytate (Romano and Kumar 2018), thus increasing the content of minerals that are bound by phytates, thereby increasing ash content.

The differences in nutrient composition of FRB may be attributed to the variation in rice bran (whole bran and defatted) used in the trials. The initial chemical composition of rice bran varies due to the differences in the rice variety used and the variation in the milling process and adulteration with the hull (Warren and Farrell 1990). Furthermore, the starter culture used, along with the length in combination with the incubation temperature, also affects the end products of fermentation.

FRB contains antioxidants in the form of phenolic compounds, flavonoids, carotenoids, and anthocyanins (Ardiansyah 2021). Several authors reported that fermentation increases the antioxidant activity (David et al. 2019; Abd Razak et al. 2015) and the bioactive compounds (Oliveira et al. 2012) of rice bran. Compared to unfermented rice bran, rice bran fermented with *R. oryzae* CCT 7560 (Ribeiro et al. 2017; Oliveira et al. 2012), *Rhizopus oligosporus* (David et al. 2019), *R. oligosporus, Monascus purpureus*, and mixture (Abd Razak et al. 2015) increase the total phenolic compounds. Cheng et al. (2016) also reported an increase of 4.2 times in the total flavonoid content of *Monascus pilosus* KCCM60084 FRB from 123 to 518 μg QE/g dry weight. The total phenolic content of plant extracts has been linked to antioxidant activity due to their redox properties, which allow them to act as reducing agents, hydrogen donors, and singlet oxygen quenchers (Cheng et al. 2016).

## 5 Effect of Fermented Rice Bran on Production

The main benefit of feeding and supplementing FRB to the diet of food‐producing animals is that it provides comparable performance to grains and cereals at a lower cost per kilogram of feed. In addition, the use of agricultural waste and by‐products as animal feeds can reduce the environmental impact of livestock production by 20% (Clark and Tilman 2017) and in combination with fermentation may preserve and improve its quality.

Several studies highlighted that the addition of a fermented diet improves production performance and the overall health of animals (Table 3). For example, Su et al. (2022) reported that the addition of 10% co‐fermented defatted rice bran with probiotics ( *B. subtilis* , *S. cerevisiae* , and *L. plantarum* ) increased the average daily gain (ADG) and the gain‐to‐feed ratio of finishing pigs. In nursery pigs, average daily gain (ADG) showed a positive response to increasing levels of FRB extract in their diets, with increased ADG (400 to 547 g/day) from days 14 to 21 (Zheng et al. 2017). This indicates that including FRB (feed or extract) can significantly enhance growth performance during the early and late stages of pig development. The antioxidative status and gut microbiota richness were enhanced considerably in finishing pigs fed diets containing FRB (Su et al. 2022). Additionally, in nursery pigs, the incidence of diarrhea was notably reduced when FRB extract was included in their diets (Zheng et al. 2017). These findings suggest that FRB not only improves growth performance but also contributes to better overall health and gut stability in pigs.

**TABLE 3:** Effect of fermented rice bran on production performance and methane production.

Animal	Substrate and inoculant (microbes/enzymes)	Inclusion rate (%)	Fermentation (h)	Results	References
In vivo					
Pig	Mixture of *B. subtilis* BSWF, *S. cerevisiae* SCWF, and *L. plantarum* CWLP	10	24 + 48	Increase ADG and G:F	Su et al. 2022
Chicken (layer)	*Lactobacillus reuteri* KCTC 1048	1	24	Increase hen‐day egg production	Kim et al. 2017
				Decrease total blood and egg cholesterol	
Chicken (broiler)	Rice bran and humic substances; *Bacillus amyloliquefaciens*	0, 5, 10, 15, and 20	72	Decreased bodyweight gain	Supriyati et al. 2015
				Improved FCR values at 15% inclusion	
Chicken (broiler)	*Aspergillus flavus*	10	96	Increase weight gain and improve FCR	Ahmad et al. 2019
Chicken (broiler)	Phytase and turmeric	5, 10, 15, and 20	120	Increase EPA, DHA, and protein in meat	Al‐Arif et al. 2020
				Decrease fat in meat	
Chicken (broiler)	*Saccharomyces cerevisiae*	8	24	Increase total weight gain and improve FCR	Azrinnahar et al. 2021
Chicken (broiler)	Rumen fluid	10	48	Increase blood albumin	Shuvo et al. 2022
				Decrease blood cholesterol	
				Improved FCR	
				Improve PER	
In vitro (sheep)	*Ligilactobacillus equi*	10	24	Increase propionate production	Manlapig et al. 2024
				Decrease methane, methane/digested DM, and total short‐chain fatty acid production	
Rumen simulation technique	*Pleurotus sajo‐caju*, *Phanerochaete chrysosporiuum*, *Trichoderma harzianum*, and *Chaetomium cellulolyticum*	100	42–44	Decrease methane production	Asiegbu et al. 1994

Abbreviations: ADG, average daily gain; DHA, docosahexaenoic acid; EPA, eicosapentaenoic acid; FCR, feed conversion ratio; G:F, gain to feed ratio; PER, protein efficiency ratio.

Ahmad et al. (2019) studied the effect of rice bran fermented with *A. flavus on* the growth performance of broiler chickens. After 42 days, a broiler‐fed diet containing FRB (96 h) has higher body weight (BW) and gain in weight over the control. Moreover, the feed conversion ratio (FCR) was improved from 2.16 to 2.04 in the treatment group. Similar findings were observed by Azrinnahar et al. (2021) who fed a 150‐day‐old straight‐run broiler for 28 days with a diet containing 8% rice bran fermented with *S. cerevisiae* (yeast); yeast and flour; and yeast, urea, and flour. Total weight gain and feed intake were significantly improved by adding FRB regardless of the addition of urea and/or flour. Accordingly, Shuvo et al. (2022) reported approximately 6.23% improvement in the total weight gain of broilers fed FRB inoculated with rumen fluid than the unfermented group. Conversely, Supriyati et al. (2015) reported that the BW of broilers fed with a diet containing 10% or more of rice bran fermented with *B. amyloliquefaciens* and humic substances was decreased. The inconsistency of results can be attributed to the differences in methodology primarily the type of microorganisms (fungi, rumen fluid versus bacteria) used during the fermentation of rice bran. Based on these results, better weight gains and final weight were observed in broilers fed with a diet containing FRB inoculated with fungi or rumen fluid. This can be attributed to the ability of fungi and microflora in the rumen fluid to produce various enzymes that free up nutrients in rice bran, thus increasing the crude protein content, reducing crude fiber and anti‐nutritional factors, which is the result of fermentation.

According to Shuvo et al. (2022), the FCR of broilers fed with a diet containing 10% rice bran fermented with rumen fluid (RF‐FRB) and 10% rice bran fermented with rumen fluid plus 5% molasses (MRF‐FRB) was significantly improved over the control diet (10% unfermented RB) at 1.72, 1.74, and 1.86, respectively. Accordingly, Azrinnahar et al. (2021) reported a 7.97% improvement in the FCR of broilers fed a diet with FRB at 8% inclusion. Ahmad et al. (2019) and Supriyati et al. (2015) also reported similar results that the inclusion of 10% and 15% FRB, respectively, in the diet of broilers improved the FCR of birds. The improvement in the FCR of broilers fed with FRB can be attributed to the increased efficiency of birds in converting feed into meat. It was observed that the total weight gain of broilers was increased without a significant rise in total feed intake, thus resulting in lower FCR values.

FRB has been reported to increase the nutritional value of meat when fed to broilers (Al‐Arif et al. 2020). They investigated the substitution effect of commercial feed with phytase‐FRB and turmeric flour on protein, fat, eicosapentaenoic acid (EPA), and docosahexaenoic acid (DHA) deposition in broiler meat. Protein, EPA, and DHA were positively influenced by the supplementation of FRB and 2% turmeric flour as observed in the increased concentration of these nutrients in the meat in response to the increasing addition of treatments in the diet. Meanwhile, fat concentration in the meat of broilers tended to be lower in treatments containing higher amounts of FRB and turmeric flour. Accordingly, the inclusion of FRB in the diet significantly reduced the cholesterol content in the blood metabolites of broilers (Shuvo et al. 2022). They reported that blood cholesterol level in the treatment groups RF‐FRB and MRF‐FRB was reduced by 22.89% and 13.09%, respectively. Kim et al. (2017) also reported lower blood and egg cholesterol content in layer chickens fed with a diet containing 1% *Lactobacillus reuteri* KCTC 1048 FRB. The reduction of fat and cholesterol in the meat and blood metabolites of the broiler can be attributed to the bioactive compounds present in rice bran and the action of the inoculant used during the fermentation process. Rice bran contains gamma‐oryzanol, which lowers cholesterol levels in the blood and exhibits antioxidant activity (Bumrungpert et al. 2019). Fermented feed also helps in reducing cholesterol biosynthesis by inhibiting the activity of 3‐hydroxy‐3‐methylglutaryl‐CoA reductase (Chen et al. 2013). Moreover, lactic acid bacteria can bind and collect cholesterol, inhibiting the absorption of bile acids in the intestine and reducing blood cholesterol levels (Hosono and Tono‐Oka 1995).

## 6 Effect of Fermented Rice Bran Ruminal Fermentation

Limited information on the effect of FRB on rumen fermentation and the few published articles focused on its ability to modulate methane production using the rumen simulation technique (RUSITEC) and in vitro fermentation methods. Asiegbu et al. (1994) found that rice bran fermented for 14 days with various fungi, including *Pleurotus sajo‐caju*, *Phanerochaete chrysosporium*, *Chaetomium cellulolyticum*, and *Trichoderma harzianum*, resulted in reduced methane production without negatively impacting DM digestibility in RUSITEC. Similarly, Manlapig et al. (2024) observed that the addition of *Lactobacillus equi* to FRB also decreased methane emissions. However, the reductions in methane production associated with rice bran fermented using lactic acid bacteria (LAB) were less pronounced compared to those achieved with fungal inoculants. This suggests that fungi may be more effective for the SSF of rice bran in terms of mitigating methane emissions.

## 7 Conclusions

This study analyzed and compared the effects of fermentation on the nutrient composition of rice bran and the performance of animals. Fermentation emerges as a viable method for enhancing the nutritional quality of rice bran by increasing the availability of active compounds, thereby improving its overall nutritional value. These positive results can be attributed to the ability of microorganisms to secrete various enzymes that break down complex nutrients in rice bran, making them more accessible for animal consumption.

Feeding FRB has been shown to improve the performance of food‐producing animals by increasing weight gain and improving efficiency. From an economic standpoint, incorporating FRB can significantly reduce feed costs compared to traditional commercial feeds. Despite this, the addition of 10% FRB is recommended, as higher inclusion rates sometimes negatively impact production performance. FRB can potentially serve as a cost‐effective replacement for expensive grains that yield optimal performance and improve overall efficiency. In summary, FRB represents an affordable source of essential nutrients, bioactive compounds, and antioxidants, collectively supporting the optimal performance and health of food‐producing animals.

## Conflicts of Interest

The authors declare no conflicts of interest.
